# Analysis of Chemical, Microstructural and Mechanical Properties of a CuAlBe Material Regarding Its Role as a Non-Sparking Material

**DOI:** 10.3390/ma17102220

**Published:** 2024-05-08

**Authors:** Romeo Gabriel Chelariu, Ramona Cimpoesu, Adrian Marius Jurca, Catalin Mihai Popa, Marcelin Benchea, Gheorghe Badarau, Bogdan Istrate, Alin Marian Cazac, Nicanor Cimpoesu, Dan-Dumitru Pintilie, Gabriel Dragos Vasilescu, Costica Bejinariu

**Affiliations:** 1 Faculty of Science and Material Engineering, Gheorghe Asachi Technical University of Iasi, D. Mangeron, 41, 700050 Iasi, Romania; romeo-gabriel.chelariu@student.tuiasi.ro (R.G.C.); ramona.cimpoesu@academic.tuiasi.ro (R.C.); gheorghe.badarau@academic.tuiasi.ro (G.B.); alin-marian.cazac@academic.tuiasi.ro (A.M.C.); nicanor.cimpoesu@academic.tuiasi.ro (N.C.); 2 National Institute for Research and Development in Mine Safety and Protection to Explosion—INSEMEX, 332047 Petrosani, Romania; adrian.jurca@insemex.ro (A.M.J.); mihai.popa@insemex.ro (C.M.P.); dragos.vasilescu@insemex.ro (G.D.V.); 3 Faculty of Mechanical Engineering, Gheorghe Asachi Technical University of Iasi, D. Mangeron, 61–63, 700050 Iasi, Romania; marcelin.benchea@academic.tuiasi.ro (M.B.); bogdan.istrate@academic.tuiasi.ro (B.I.); 4 Doctoral School, University of Petrosani, Universitatii Street 22, 332006 Petrosani, Romania; 5 Academy of Romanian Scientists, Ilfov 3, 050044 Bucharest, Romania

**Keywords:** non-sparking material, CuAlBe, microstructural and mechanical properties

## Abstract

We developed and analyzed a novel non-sparking material based on CuAlBe for applications in potentially explosive environments. Using a master alloy of CuBe, an established material for anti-sparking tools used in oil fields, mines, or areas with potentially explosive gas accumulations, and pure Al, we used an Ar atmosphere induction furnace to obtain an alloy with ~10 wt% Al and ~2 wt% Be percentages and good chemical and structural homogeneity. The new material was tested in an explosive gaseous mixture (10% H_2_ or 6.5% CH_4_) under extremely strong wear for 16,000 cycles, and no hot sparks capable of igniting the environment were produced. The material was used in the form of hot-rolled plates obtained from melted ingots. The experimental results reflect the use of a suitable material for non-sparking tools. This material has good deformability during hot rolling, abnormal grain growth during deformation under heat treatment and special thermo-mechanical processing, and no high chemical composition variation. Additionally, there are slightly different corrosion resistance and mechanical properties between the melt and hot-rolled state of CuAlBe material. Through hot rolling, the material’s corrosion resistance increased, reducing the chances of generating sparks capable of causing explosions.

## 1. Introduction

The need to reduce the frequency of explosions and instantaneous fires in the workplace can be explained by both humanitarian and economic considerations [1]. The humanitarian considerations are obvious: explosions and fires can cause extremely serious injuries and deaths. The economic considerations emerge from studies devoted to the real costs of accidents, which show that an improvement in risk management (for health and safety) can considerably increase company profits. Protection against explosions is of particular importance for safety because explosions endanger the lives and health of workers due to the uncontrolled effects of flames and pressure, the presence of harmful reaction products, and the consumption of oxygen from the ambient air breathed by the workers [1,2]. An explosion occurs when a fuel mixed with air (containing a sufficient amount of oxygen) reaches the explosive limits in the presence of an ignition source [3]. In the event of an explosion, workers are exposed to risks resulting from uncontrolled ignition and pressure phenomena, such as thermal radiation, flames, shock waves, and debris projection, as well as the presence of harmful reaction products and the depletion of oxygen, indispensable for breathing, in the air.

The most well-known and widely used anti-sparking alloy for obtaining active tools is CuBe. It is used to manufacture screwdrivers, hammers, shovels, pliers, etc. [3,4]. More complex gears are embedded and encapsulated in hermetically sealed shells to separate them from the potentially explosive material enclosed within, and this approach substantially increases the cost of more-complex devices. To obtain metal parts for these gears, the authors propose using CuAlBe alloy, which satisfies both the processing requirements and mechanical characteristics [5,6].

Bronzes have good sliding properties; therefore, they are widely used in gears. They are extremely stable and give a machine optimal working properties, but they also present disadvantages: their weight and high price. Aluminum bronzes are used as materials for the following: gears, bearings, and bushings. Aluminum bronzes possess innate resistance to drops. They are used when resistance to rust, erosion, and waterproofing is required. Aluminum is the main reinforcing element and is normally present at between 8% and 13%wt. At the higher end of the range, hardness figures of 30–44 HRC are possible. However, at these very high hardnesses, ductility is reduced to 1% [4]. The friction tests for the approval of anti-spark protection materials intended for use in explosive atmospheres are conducted under laboratory conditions and assume that sparks are generated by accidental or technological friction in explosive gas mixtures [5].

In this work, two alloys with new compositions from the CuAlBe system were tested in different states (cast, heat-treated, or hot-rolled) in an effort to obtain anti-sparking elements such as gears composed of new materials.

## 2. Experimental Materials

In contrast to the CuBe alloy used to manufacture tools for environments with potentially explosive atmospheres, the addition of Al improves formability, deformability, and corrosion resistance properties. By controlling the component phases and microstructure, spark-causing situations can be avoided, thus limiting the possibility of an explosion. The microstructures and surfaces of the materials subjected to wear tests were investigated using light optical microscopy (LOM, Zeiss Imager Axio a1M, Erfurt, Germany) and scanning electron microscopy (SEM, VegaTescan LMH II, Brno-Kohoutovice, Czech Republic). Chemical composition and phases were evaluated using a Bruker X-ray energy-dispersive detector, X-Flash 6-10, Billerica, MA, USA, and XRD equipment, X’Pert Panalytical, Enigma Business Park, Grovewood Road, Malvern, UK. Corrosion resistance (open-circuit potential, linear and cyclic potentiometry) and surface aspects were analyzed in a saltwater electrolyte solution. Mechanical properties such as microhardness and the friction coefficient were determined using UMT. Severe wear tests showed the excellent behavior of these alloys in an explosive atmosphere in terms of generating sparks capable of causing explosions.

The experimental alloys were obtained from high-purity Cu (99.995%), CuBe master alloy (Be 4%wt), and high-purity aluminum (99.99%).

Based on a literature review and the known alloys CuBe (1–2%Be) for anti-sparking applications and CuAl (approx. 10% Al) for various structural elements, we aimed to obtain a CuAlBe alloy to replace 1–2% of the Al proportion with Be.

**Figure 1:**
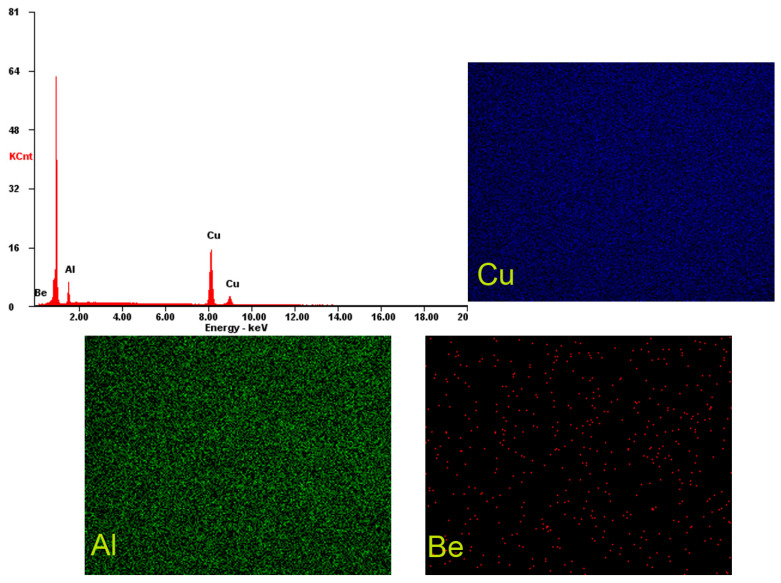
EDS spectrum of the CuAlBe sample and mapping of the main elements (Cu, Al, and Be).

The charge was melted in an induction furnace in a ceramic crucible and melted again at 1100 °C in a laboratory electric furnace 5 times to ensure the chemical and structural homogeneity of the alloy and eliminate casting defects; detailed images are given in Figure 1, mapping the elements over a 1 mm^2^ area. The average chemical compositions of the alloys after 3 determinations on a 1 mm^2^ surface are shown in Table 1.

**Figure 2:**
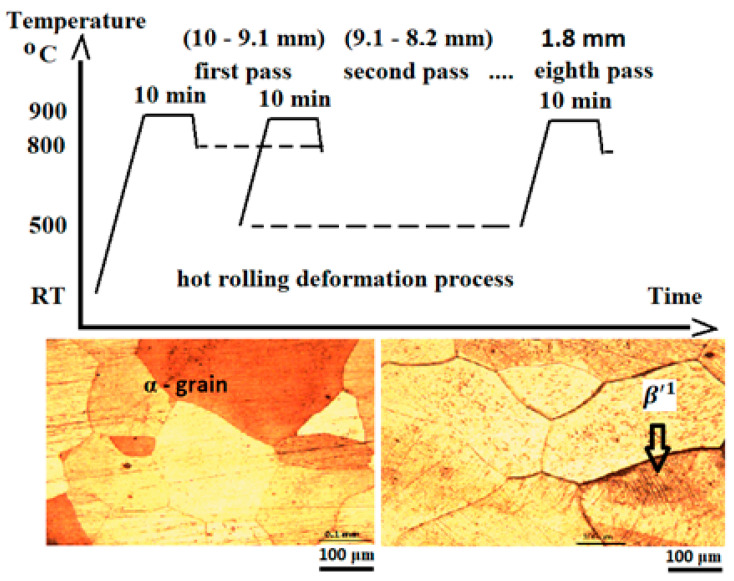
Schematic diagram of CuAlBe hot-rolling process and microstructure (optical microscopy) before and after deformation.

The experimental alloys were heat-treated by heating them to 850 °C and cooling them in water, and they were also hot-rolled by heating them to 900 °C and rolling them according to the scheme in Figure 2, thus yielding ingots with a diameter of 10 mm and a length of 100 mm and plates with a thickness of 1.5–2 mm and a length of 180 mm.

Usually, bronzes are easy to roll using both cold and hot processes, depending on their chemical composition. For CuAl alloys, it is recommended that the hot-rolling process be performed in a heating range between 800 °C and 950 °C [6,7].

Beryllium bronzes are technical alloys containing 2–3% Be and exhibit the following phases: an α-solid solution based on the substitution of Be in Cu, with a CFC network, and γ solid solution based on CuBe compound, which is hard and brittle. Because of the variation in the Be solution in Cu with temperature, these bronzes can be tempered from 800 °C to the α-structure, at which point they exhibit a particular plasticity. They can be precipitation-hardened after aging at 300–350 °C. These are easily deformable, elastic, corrosion-resistant, weldable, and anti-spark bronzes. They are used for mine hammers and chisels, springs, diaphragms, watchmaking parts, etc.

Beryllium is an alloying element that positively and spectacularly changes the physico-mechanical properties, resistance to cavitation corrosion, refractoriness, and operational reliability of Al bronzes. It also adds antimagnetic and anti-sparking properties to bronzes containing Al [8].

Microstructural analysis was performed using a Zeiss optical microscope (Zeiss, Jena, Germany) with a MotiCam optical camera at room temperature (≈25 °C). For microstructural analysis, the samples were sanded with SiC paper to a grain size of 2400 mesh and mechanically polished using an aqueous alumina slurry (1–0.3 μm). A chemical attack was performed using an aqueous solution of iron chloride (FeCl_3_) for 10 s [9].

A polycrystalline austenite-like structure of the alloy at room temperature and narrow stress-induced martensite zones after rolling were observed for the sample with more Be. Regarding the structural aspects, an abnormal grain growth after rolling, which has also been reported in other papers [10]; an orientation of the structure along the rolling direction; and the appearance of stress-induced martensite β′1 were observed (Figure 2).

## 3. General Testing Procedure

This procedure describes how to perform the approval test for spark protection materials intended for use in explosive atmospheres; it is part of the group of tests used for material and equipment performance verification.

This procedure is applicable in laboratories for the acceptance testing of spark protection materials intended for use in potentially explosive atmospheres.

The normative references used in this procedure were extracted from the following standards: the Romanian standard STAS 10449-86 (Electrical Equipment for Potentially Explosive Atmospheres “Impact and friction tests”) and the standard SR EN ISO/IEC 17025:2018, “General requirements for the competence of testing and calibration laboratories” [11,12].

An explosive mixture consisting of air containing 6.5% CH_4_ is used to test materials for equipment in Groups I and II A. An explosive mixture composed of air and 10% H_2_ is used for material testing. A friction test, impact test, or both may be performed to verify whether hot sparks capable of producing a fire are likely to occur when a particular material is used in an explosive environment under certain conditions. For gear wheels, the determining factor for spark occurrence is the friction process.

**Figure 3:**
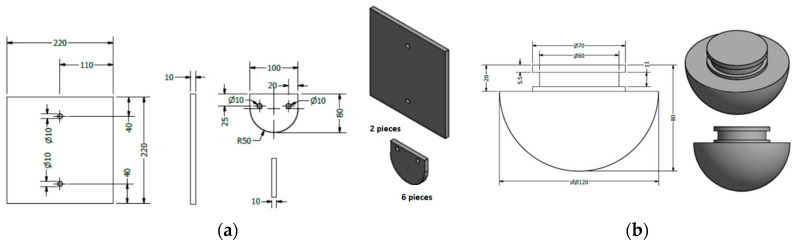
The metallic samples for testing (specimens) made from the pairs of materials subjected to the test. The dimensions are presented in mm for (**a**) friction tests and (**b**) impact tests.

For the friction test, the following materials are required: a special stand equipped with a rotating disk driven by an electric motor; a test chamber with dimensions of 1000 × 1000 × 1000 mm; samples made from material pairs with the dimensions shown in Figure 3a and a movable support for fixing the plate that is capable of translation parallel to the axis of the rotating disk; a test sample with a rounded friction surface, fixed to the rotating disk; and an automated device for creating an explosive mixture, containing the dosing installation and oxygen analyzer. The oxygen analyzer has a measuring range of 0 to 100% O_2_. An oxygen cylinder and an ignition source (voltage generator + spark plug) must also be used to ignite the mixture only if the friction process has not produced hot sparks so that the mixture is not released into the atmosphere. Metal test samples (specimens) made from the material pairs to be tested with the corresponding dimensions (in mm) are shown in Figure 3a.

The impact tester uses a stand composed mainly of a table providing sufficient energy for the test; a digital system for determining drop height and calculating impact energy; the test sample; a rusted steel plate; an explosion chamber (the outer wall is open and covered with a plastic sheet to allow the release of the explosion pressure); a device allowing the longitudinal and transverse movement of the rusted plate; and explosive-mixture-generating equipment consisting of a dosing installation and the oxygen analyzer. The oxygen analyzer has a measuring range of 0 to 100% O_2_.

In addition, an oxygen cylinder and an ignition source (voltage generator + spark plug) are needed to ignite the mixture only if the impact process has not produced hot sparks, thus failing to release the mixture into the atmosphere.

The results of the impact test, where the test material sample has the shape presented in Figure 3b (showing whether the test material passed or failed the test), are described according to the occurrence or non-occurrence of ignition in the first 10 tests in the explosive mixture or the number of ignitions in the next 32 tests in the oxygen-enriched or double-impact energy explosive mixture. The impact test was performed under laboratory conditions and consisted of the simulation, on special stands, of the spark formation process generated by accidental or technological impacts. The simulation was conducted on a special test stand, on which an impact was generated between the vertically inclined rusted plate and the sample to be tested. The impact energy was determined by using a digital system for determining the falling height and calculating the impact energy. To calculate the impact energy, the digital system uses the following formula: E = m × g × h [J]; here, m is the falling mass [kg], g is gravitational acceleration [m/s^2^], and h is the falling height [m]. The weight shape is chosen according to the application of the material used in the construction of the equipment [13,14].

Samples of the rolled material, in the form of plates, were used to determine several surface characteristics using a Cetr-Umt2 tribometer [8,15].

Experiments were performed on parallel-sided samples with dimensions of 50 × 20 × 2 mm. Scratch tests were performed on the polished surfaces of the samples (grains and grain boundaries). The experiment was performed by applying an increasing force from 1 to 25 N over a 10 mm distance at a 1 mm/s speed on the etched area of the samples.

Corrosion resistance was evaluated using linear and cyclic potentiometry tests using a VoltaLab 21 electrochemical system (PGP201-Economic Potentiostat) equipped with a three-electrode cell and VoltaMaster 4 data-acquisition-and-processing software. Samples subjected to an electric current in a saline electrolyte medium were mechanically polished and washed with distilled water before the experiment. A saline solution (NaCl 3.5%) at room temperature was used as the electrolyte solution [16].

Linear polarization curves were plotted, showing the electrode potential rate variation, dV/dt = 0.5 mV/s, and cyclic polarization curves with a 10 mV/s rate. The surface conditions were investigated using optical microscopy, scanning electron microscopy and energy dispersive spectroscopy. The experiments were conducted in compliance with occupational health-and-safety laws and regulations to eliminate all risks and hazards that may affect human resources during the experimental procedures [17].

## 4. Experimental Results and Discussions

At high temperatures, these alloys exhibit a stable β-phase with a disordered structure that can be preserved at low temperatures through rapid cooling. The disordered β-phase arranges into a DO3 structure during cooling at a lower solidification rate; it decomposes into γ2 and α phases with a lower or higher aluminum content. The martensitic transformation of the β-phase into 18R can be induced spontaneously via cooling or mechanically via tension.

### 4.1. XRD Analysis

**Figure 4:**
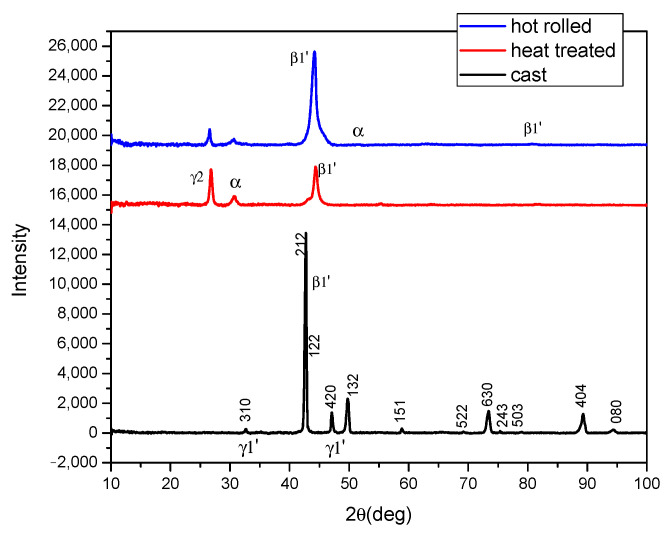
XRD patterns of CuAlBe alloy in different states: cast, heat-treated, and hot-rolled.

For the cast state, the main phase is martensitic (Figure 4) and composed of the orthorhombic phase (18R-β1′) with small fractions of the monoclinic system (2H-γ1′) at 2ϴ angles of 33 and 47 [7].

Following the heat treatment (Figure 4), in addition to a large, straight martensite peak at a 2ϴ angle of about 44 typical for the β1′ phase (Cu_3_Al) due to precipitation, degradation of the martensitic transformation is visible for the hot-rolled sample, primarily due to heating at 900 °C before plastic deformation by the precipitation of γ2 (Cu_9_A_l4_) compounds and the α-phase (lower Al content) by the appearance of 2ϴ peaks at 27 and 31.

After being rolled, the alloy (Figure 4) was found to be in a single-crystal state with a low degree of polycrystallinity, not much of an amorphous phase, and few defects. The only large peak that appeared is the one with the (120) plane indicating a martensitic β1′ phase. Next to this peak, to its right, appears another α peak (200) around a 2ϴ angle of 50 and another also of martensitic β1′ (042) around a 2ϴ angle of 80 γ [18,19].

### 4.2. Electro-Chemical Corrosion Resistance

Cu-based alloys generally exhibit high corrosion resistance and are used in many applications in highly corrosive environments. The addition of aluminum improves their corrosion resistance, especially in saline environments, in chlorides, as it contributes to increasing the protective character of the composite layers [20,21].

Studies in the literature show that the corrosion process occurs via dealumination through the preferential removal of the aluminum-rich or aluminum phase from the alloy [22,23].

**Figure 5:**
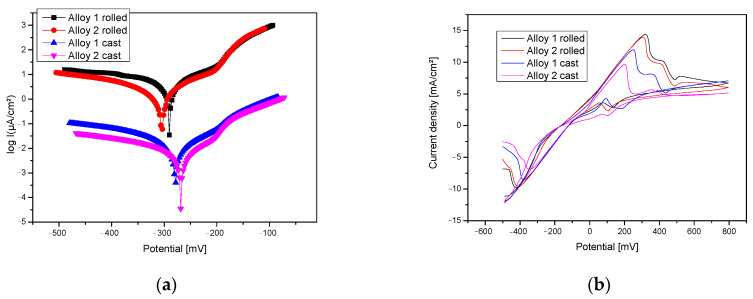
Linear and cyclic potentiometry results. (**a**) Tafel plot and (**b**) current density vs. potential.

The different behaviors of CuAlBe alloys in chloride solutions are primarily caused by their microstructure and component phases. In the γ2-phase samples, dissolution of precipitates occurs, thus protecting the matrix from the solid solution and reducing its desalination process. On the other hand, precipitates have a higher Cu content, hence their being marked as α, and show higher corrosion stability [23]. Linear potentiometry (Tafel curves in Figure 5a) shows similar behaviors of the four samples, grouped two by two according to their condition: cast, hot-rolled, and heat-treated. The repassivation potential is in the range 180–200 mV/SCE for the cast samples and 160–180 mV/SCE for the rolled samples.

**Figure 6:**
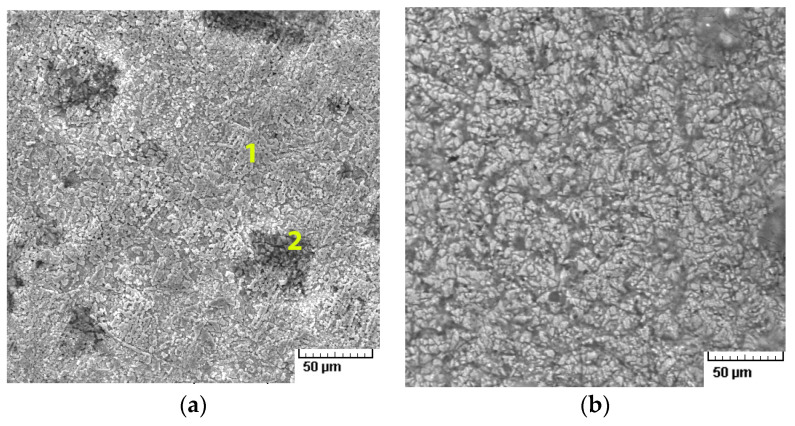

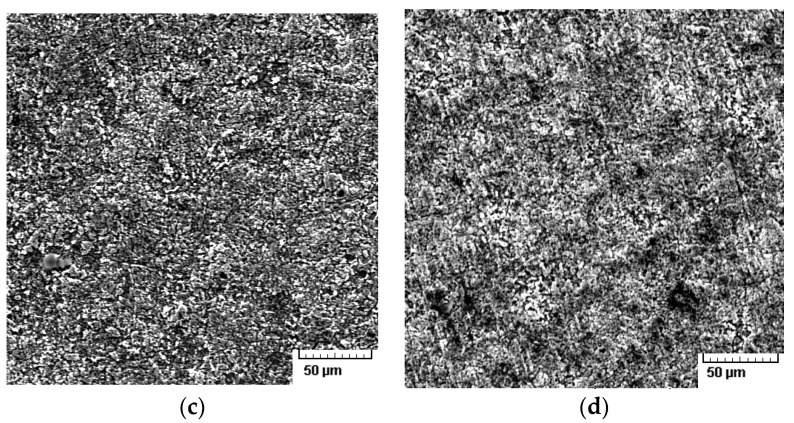
SEM images of the CuAlBe surfaces after electro-chemical corrosion tests (**a**,**b**) in cast state and (**c**,**d**) hot-rolled state.

The optical observations on the surface were confirmed via SEM images and showed the formation of a compound layer as a result of the interaction between the alloy and the electrolyte (Figure 6a–d).

The main anodic reaction is the formation of copper and aluminum oxides as well as the hydroxides of these elements, and the reduction of oxygen is the main cathodic reaction. Alumina can be generated on the surface through Al complexation by existing chlorides and a subsequent hydrolysis step. Table 2 shows the process parameters recorded during the electrochemical corrosion resistance test after the open-circuit potential analysis conducted for 60 min. The cyclic potentiometry results, recorded and interpreted as a function of the potential current density (Figure 5b), show the pitting corrosion behavior of the alloy’s surface. The curves also indicate the passivation intervals of the surface through the formation of copper oxides, which generally form a protective layer on the surface.

Although no variation in the corrosion resistance of the CuAlBe alloys as a function of the percentage of Be was reported, a large difference in behavior was observed between CuAlBe samples with different phases in the composition. Hence, the presence of other phases in addition to β will decrease the corrosion resistance of these alloys through the formation of local micro-stacks between the phases.

For Cu-Al alloys, in addition to the dissolution and oxidation of Cu, the same corrosion process occurs for Al. Thus, in addition to the Cl compound, Al salts, such as AlCl and Al(OH)_2_Cl, were incorporated on the surface.

It has been reported in the literature that among the first corrosion products of Cu-based alloys in chloride environments is CuCl, leading further to the formation of Cu_2_O, copper oxide, which proceeds to oxidize to CuO at higher oxidation potentials [24].

**Figure 7:**
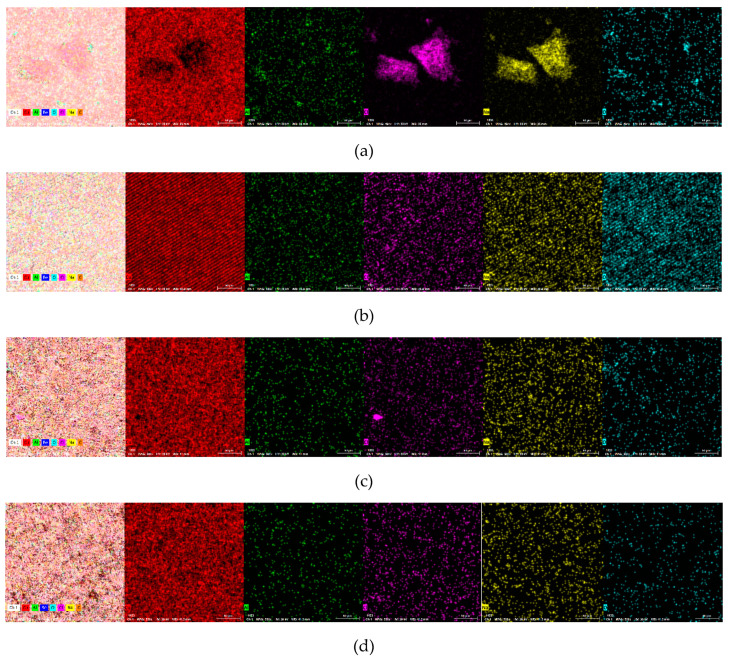
Elemental distribution on CuAlBe surfaces (Cu, Al, Cl, Na, and O) after corrosion tests for (**a**) Alloy 1 (cast), (**b**) Alloy 2 (cast), (**c**) Alloy 1 (hot-rolled), and (**d**) Alloy 2 (hot-rolled).

In addition, the presence of a CuCl_2_ compound was identified on the surfaces of the Cu alloys subjected to cyclic potentiometry tests. The surfaces of the alloys showed dealumination that transformed the granular structure into a porous Cu-rich matrix. The detection of Al oxides on the surface corresponds to corrosion compounds formed in areas where the β-phase was dissolved. All cases (Figure 6a,d) show advanced surface corrosion due to the concentrated electrolyte solution used, more specifically a localized one-phase type. The presence of compounds based on oxides, carbides, or chlorides can also be observed on the surface (Table 3 and Figure 7).

The surface structure of Alloy 1 (Figure 6a) presented (β + γ2) phases after the anodic polarization test in 3.5% NaCl [16], showing pronounced Al depletion of the surface (zone 1) and containing some corrosion products (zone 2: Al_2_O_3_, CuO, Cu_2_O, and even NaCl deposits). On the matrix (zone 1), pits produced by severe dealumination can be observed, which contain a porous product with a copper content of up to 87% wt. In other areas of the sample, the Al_2_O_3_ film could not be observed on the precipitates, but a copper-rich pore product was detected.

The presence of corrosion compounds based on Al, O, Cl, Na, or C on the surfaces of the alloys was confirmed by EDS analysis. The compounds that could form on the surface are Al_2_O_3_, CuO, Cu_2_O, and even NaCl deposits. Be was also detected, indicating the formation of Be-based corrosion compounds. The open-circuit potential values show that both alloys are prone to spontaneous corrosion when in contact with an electrolyte solution and especially when in direct ion exchange. Surface spectrometric analysis showed a more resistant coating of compounds for the cast alloys compared with the rolled ones, with a lower percentage of Cu identified on the surface.

On account of the difficulties in detecting Be, which are usually encountered with most spectroscopic techniques, it was observed that because of the state of the formed compounds, Be could not be quantified, even though in the distribution analysis, the Be is present on the surface, but in small quantities and areas. The elemental distributions of elements on the CuAlBe surfaces after the corrosion tests are shown in Figure 7 for Cu, Al, Cl, Na, and O, only because Be was not identified on the cast and corroded samples, and the presence of C in Table 3 may be a confusion or misinterpretation of the EDS detector.

Salts, NaCl, or oxides, especially CuO-based ones, were observed on the corroded surfaces (Figure 7). Most oxides detached from the surface and passed into the electrolyte solution.

### 4.3. Scratch Tests Results

**Figure 8:**
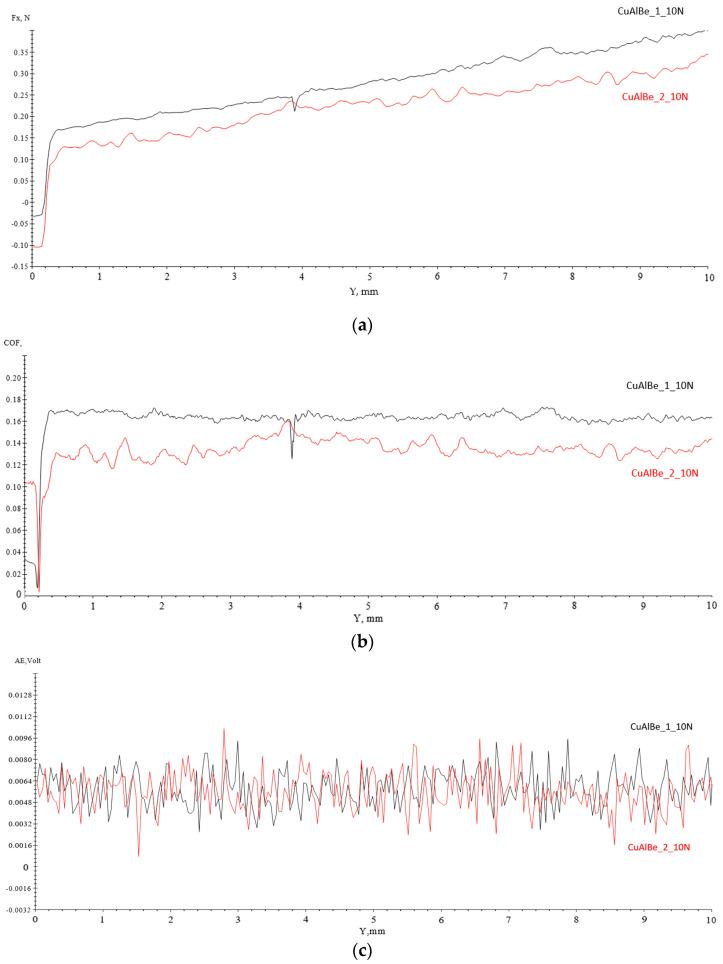
Variations of the alloy surface reaction force in the cast state in (**a**) of the coefficient of friction (CoF) in (**b**) and of the acoustic emission (AE) in (**c**).

In terms of mechanical behavior, Cu, CuBe, and most C-based alloys are elasto-plastic materials. At low strain, the specimens exhibited a linear elastic zone, and for loads higher than their yield strength, they underwent irreversible plastic deformation with wide strain limits. The scratch resistance experiments for the two specimens show similar results (Figure 8 and Table 4) independent of the percentage of Be used. The recorded variations were Fx (material strength with respect to scratch force), CoF (friction coefficient), and AE (acoustic emission, representing the occurrence of acoustic (elastic) ripple propagation in solid materials) over a 10 mm distance.

Variations in chemical composition (percentage of Be; Table 1) and material processing via hot rolling do not reveal large differences in the behavior of the surface mechanical properties [3,25].

CoF exhibits small surface fluctuations that may result from grain boundaries, stress-induced martensite zones, or various metal compounds with a lower friction coefficient than the copper-based solid solution [26]. Stress-induced martensite (M) zones or residual martensite are softer in terms of hardness and stiffness than the rest of the material in the austenitic state. The friction coefficient is slightly higher for Alloy 1 (Figure 8b) due to the lower percentage of Be in the alloy and reduced microhardness caused by the very small number of intermetallic compounds containing Be.

Based on the acoustic emission variations (Figure 8c), no consequences of crack propagation or plastic deformation of the material due to aging, temperature gradients, or external stresses were observed [3]. According to the values determined from the scratch test (Table 4), a higher friction coefficient of sample 2 for the alloy with a higher mass percentage of Be was observed. This shows that the presence of Be in a higher percentage favors the formation of stress-induced martensite, a softer phase than β austenite, which leads to an increase in the friction coefficient of the alloy. The acoustic emission values were very similar, and the shape of the variation curves confirms the polycrystallinity of the alloys and the existence of different phases and/or components with different stiffnesses [27].

### 4.4. Results of Wear Tests for Hot Spark Generation

CuAlBe alloys can exhibit pseudoelastic behavior at room temperature [28,29]. The hysteresis curve is associated with the dissipation of mechanical energy into heat. Because of these characteristics, these alloys can be used as external stress absorbers. The anti-spark character of these alloys could result from their high capacity to absorb mechanical stresses and therefore inability to generate hot sparks. Table 5 presents the main conclusions of the wear resistance tests conducted in a potentially explosive atmosphere.

The sparks resulting from the friction process are considered harmless (i.e., the tested pair of materials passed the test) if the following conditions were met:

- There was no ignition during the first 16,000 friction cycles in the explosive mixture;
- No more than eight ignitions occurred during the next 16,000 friction cycles in the explosive mixture enriched with oxygen up to 25%.

In conclusion, given the test results presented above and the testing conditions, the evaluated materials can be considered non-sparking.

**Figure 9:**
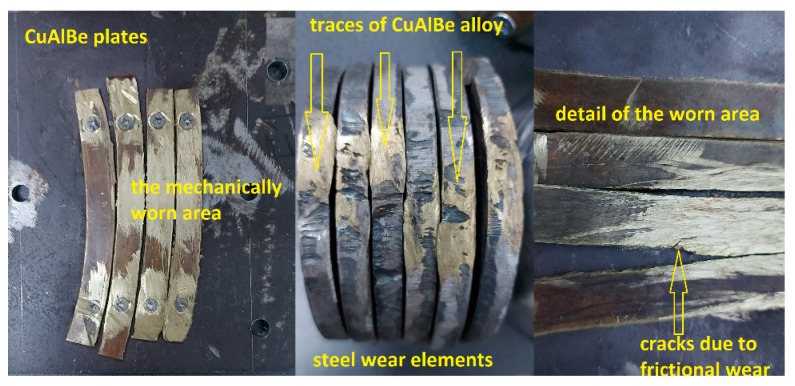
State of the surfaces of the experimental alloy plates after the wear test.

Figure 9 shows the state images of the experimental alloy in the form of plates after being subjected to frictional wear for 16,000 passes.

The contact intensity was observed by passing the CuAlBe material over steel wear pads, as shown in the middle image in Figure 9. In addition to the passage of the lower-hardness Al–bronze material on the steel used for friction, several cracks on the material plates can be observed in the right-side image in Figure 9 [30,31]. These results were derived from Alloy set 1 with the predominant austenite phase. Therefore, the Alloy 2 plates behaved similarly in terms of the production of explosion-initiating sparks and were similar. The samples subjected to severe wear were in a rolled and heat-treated state, and regardless of the component phases, no quantity was capable of producing explosion-initiating sparks [32].

**Figure 10:**
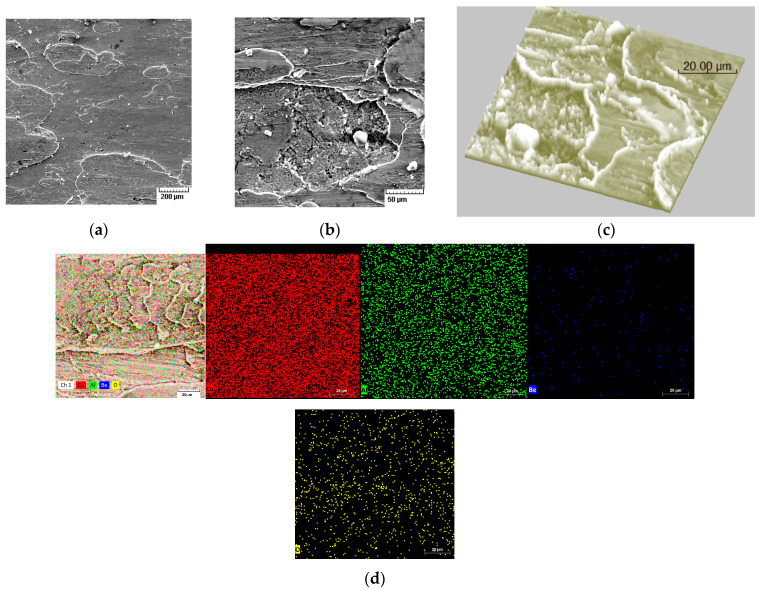
SEM images of the surface after the wear test: (**a**) 500×, (**b**) 1000×, (**c**) 1500×—3D, and (**d**) elemental distributions on the worn area.

Figure 10 shows the surface conditions of the experimental alloys after wear. Detachment of the material from the surface and the formation of corrosion oxides on the employed surface can be observed.

Sparks resulting from the friction process were considered non-hazardous (i.e., the pair of materials tested passed or failed the test) if no ignition occurred during the first 16,000 friction cycles in the explosive mixture or no more than eight ignitions occurred during the next 16,000 friction cycles in the explosive mixture enriched up to 25% with oxygen. It can be noticed that the alloy was exfoliated from the surface in several layers for different stresses depending on the number of passes performed (Figure 10c). Oxidation of the surface frequently occurred, without having a decisive influence on the spark production capacity of the material (Figure 10d).

## 5. Conclusions

Following the experimental tests, the following was observed:

- In addition to serving as dedicated materials for non-sparking metallic parts, CuAlBe alloys can be used as non-sparking materials for various industrial applications involving potentially explosive atmospheres;
- Using alloys in a rolled state without heat treatment does not affect the type of sparks produced;
- Following the hot rolling of the alloys, their corrosion resistance increases at least eight times, reducing the chances of producing sparks capable of causing explosions;
- The small percentage variation of Be in the tested alloy does not significantly affect its corrosion resistance or mechanical properties;
- The severe wear experiments performed over 16,000 cycles and including material transfer did not produce sparks causing explosions, regardless of the component phases of the alloy.

## Figures and Tables

**Table 1:** Chemical compositions of experimental CuAlBe alloys.

Chemical Composition	Cu %		Al %		Be %		Others %
	wt	at	wt	at	wt	at	wt%
Alloy 1	90.5	78	8.5	17.2	0.8	4.8	0.2
Alloy 2	91.0	79.2	8.4	17.3	0.4	2.5	0.2

St. dev.: Cu: ±1.5; Al: ±0.5; Be: ±0.05.

**Table 2:** Electro-chemical corrosion test parameters.

Alloy	Corrosion Process Parameters					
	E (I = 0) (mV)	i_corr_ (µA/cm)	Rp (kohm/cm)	v_corr_ (μm/an)	−β_c_ (mV/dec)	β_a_ (mV/dec)
Alloy 1, cast	−278.9	31.6	1.86	436.0	378.0	122.2
Alloy 2, cast	−268.5	30	3.77	414.5	725.2	123.8
Alloy 1, rolled	−290.3	3.73	5.84	51.45	314.4	122.3
Alloy 2, rolled	−306.1	3.49	14.53	48.11	379.6	83.0

**Table 3:** Chemical composition of cast and rolled Alloys 1 and 2.

Surface		Alloy 1 Cast	Alloy 2 Cast	Alloy 1 Hot-Rolled	Alloy 2 Hot-Rolled	EDS Error %
Cu %	wt	79.79	68.05	87.39	86.45	1.5
	at	53.24	34.45	56.68	55.64	
Al %	wt	0.55	0.89	0.32	0.17	0.1
	at	0.86	1.06	0.48	0.26	
Be %	wt	-	-	3.23	2.69	0.01
	at	-	-	14.75	12.22	
Na %	wt	9.02	4.88	1.23	2.01	3.93
	at	16.65	6.82	2.21	3.58	
C %	wt	6.13	9.82	6.91	7.51	10
	at	21.65	26.32	23.70	25.60	
Cl %	wt	2.98	1.38	0.16	0.18	1.2
	at	3.57	1.26	0.18	0.20	
O %	wt	1.54	14.98	0.77	0.97	0.5
	at	4.04	30.11	1.98	2.50	

St. dev.: Cu: ±0.2; Al: ±0.05; Be: ±0.05; Na: ±0.6; C: ±2; Cl: ±0.25; O: ±0.25.

**Table 4:** Mechanical properties of the alloys in the cast state (average).

Materials	F_x_ [N]	AE [V]	CoF	F_f_ [N]	−Fz [N]
CuAlBe_1_10N	0.043	0.0058	0.179	1.043	5.24
CuAlBe_2_10N	1.71	0.006	0.274	1.713	5.47

**Table 5:** Mechanical properties of the alloys in the rolled state (average).

Nr. Crt.	Sample Type	Explosive Mixture (10% H_2_) or 6.5%CH_4_	Pair of Materials	Occurrence of Ignition during the First 16,000 Friction Cycles in Explosive Mixture (YES/NO)	Occurrence of Ignition during the First 16,000 Friction Cycles in Explosive Mixture Enriched with O_2_ up to 25% (YES/NO)
1.	Samples CuAlBe_1	10% H_2_	F1-CuAlBe_1 and steel	NO	NO
2.	Samples CuAlBe_2	10% H_2_	F1-CuAlBe_2 and steel	NO	NO

## Data Availability

Data are contained within the article.
